# Effects of Neuromuscular Electrical Stimulation and Blood Flow Restriction in Rehabilitation after Anterior Cruciate Ligament Reconstruction

**DOI:** 10.3390/ijerph192215041

**Published:** 2022-11-15

**Authors:** Doo-Hwan Kong, Won-Sang Jung, Sang-Jin Yang, Jin-Goo Kim, Hun-Young Park, Jisu Kim

**Affiliations:** 1 Department of Sports Medical Center and Sports Medical Research Institute, Seoul Paik Hospital, Inje University, 9 Mareunnae-ro, Jung-gu, Seoul 04551, Republic of Korea; 2 Department of Sports Medicine and Science, Graduate School, Konkuk University, 120 Neungdong-ro, Gwangjin-gu, Seoul 05029, Republic of Korea; 3 Physical Activity and Performance Institute, Konkuk University, 120 Neungdong-ro, Gwangjin-gu, Seoul 05029, Republic of Korea; 4 Department of Health and Exercise Management, Tongwon University, 26 Gyeongchung-daero, Gonjiam-eup, Gwangju-si 12813, Republic of Korea; 5 Department of Orthopedic Surgery and Sports Medical Center, Myong-Ji Hospital, 55 Hwasu-ro 14beon-gil, Deogyang-gu, Goyang-si 10475, Republic of Korea

**Keywords:** anterior cruciate ligament reconstruction, balance, blood flow restriction exercise, muscular function, neuromuscular electrical stimulation, rehabilitation

## Abstract

The present study aimed to examine and compare the effects of a rehabilitation exercise (RE) using neuromuscular electrical stimulation (NMES) and blood flow restriction (BFR) on muscle function and knee functional abilities in patients who underwent anterior cruciate ligament reconstruction (ACLR). A total of 45 patients who underwent ACLR (28.76 ± 0.8 years; 34 males and 11 females) were retrospectively divided into three groups: control (CON, n = 15), NMES (n = 15), and BFR (n = 15). All participants carried out the RE program for 60 min, thrice a week for 12 weeks. The Lysholm score, International Knee Documentation Committee (IKDC) subjective score, thigh circumference at 5 cm from the knee joint, Y-balance posterior medial, and lateral significantly increased in all groups via intervention (*p* < 0.05). However, NMES showed a higher thigh circumference at 15 cm from the knee joint than CON via intervention (*p* < 0.05), and the strength and endurance of quadriceps femoris and hamstrings and Y-balance anterior showed a significant increase via intervention in NMES and BFR compared with CON (*p* < 0.05). In conclusion, we confirmed that RE using NMES and BFR effectively enhances muscle function and balance in ACLR patients.

## 1. Introduction

In the field of sports medicine, anterior cruciate ligament (ACL) surgery due to knee injury is frequently performed, and quadriceps femoris weakness is generally observed [1]. The muscle weakness of the quadriceps femoris muscle due to ACL injury is caused by muscle atrophy and a nerve-suppressive reaction that blocks muscle activation of the quadriceps femoris [1,2,3]. Atherogenic muscle inhibition is related to joint swelling, inflammation, pain, joint relaxation, and structural damage that occur after knee injury or knee surgery [2,3]. ACL injury, in particular, causes more muscle atrophy through a mechanism that inhibits quadriceps femoris activity [1]. Because quadriceps femoris weakness caused by a knee injury and surgery exacerbates dynamic knee stability [4,5] and functional ability [6] and increases the risk of arthritis [7] and knee joint re-injury [8], urgent and effective medical treatment is required.

Even if the overall rehabilitation process is performed after ACL surgery, the muscle function of the quadriceps femoris does not fully recover to the pre-ACL surgery state [9,10]. After ACL surgery, quadriceps femoris weakness is associated with changes in movement patterns [11], decreased muscle function [12], increased risk of re-injury [13], and thinning of the articular cartilage of the femur [14]. In particular, muscle atrophy and muscle weakness occurring after ACL injury and surgery are important issues, and such muscle atrophy and muscle weakness can persist for many years after ACL surgery [15]. A previous study reported that the cross-sectional area of the quadriceps femoris between the ACL surgical site and the non-surgical site was 18% or more even after six years [16]. Therefore, a rehabilitation exercise modality to restore the muscle function of the quadriceps femoris more effectively after ACL surgery is very important.

Neuromuscular electrical stimulation (NMES) is a method using large electrodes and multiple current paths, and it is a method of complexly delivering current using multiple paths rather than the traditional form of electrical stimulation [17]. That is, the electrical stimulation generated by the NMES equipment stimulates the motor nerve of the muscle through multiple current paths to induce continuous contraction and relaxation of the muscle, thereby inducing muscle improvement, that is, muscle hypertrophy [17,18]. Previous studies have reported that NMES minimizes quadriceps femoris atrophy [18], increases the strength of muscle contraction during exercise [19], and enhances muscle function [20]. Additionally, the repetitive electrical stimulation of skeletal muscle using NMES shows physiological improvement such as an increase in cross-sectional area [21], suppression of a decrease in muscle mass [22], and an increase in the motor unit of neuromuscular muscle [23]. By inducing hypoxic conditions in local muscle tissue via resistance exercise with reduced oxygen supply, BFR stimulates chemoreceptor types III and IV of muscle afferents [24,25], and it is thus an effective modality for inducing the recruitment of more fast muscle fibers to improve muscle function and induce muscle hypertrophy [26]. A previous study reported that the combination of BFR and low-intensity resistance exercise can induce the effect of high-intensity resistance exercise, which leads to an enhancement in muscle function and hypertrophy [27]. As summarized above, NMES and BFR are suitable for the rehabilitation exercise modality of patients undergoing ACL reconstruction, and it is very important to evaluate their clinical effectiveness. In addition, studies comparing the effectiveness of general rehabilitation exercise, NMES, and BFR to patients who have underwent ACL reconstruction are lacking.

Therefore, the purpose of the present study was to examine and compare the effects of the rehabilitation exercise modality using NMES and BFR vs. general rehabilitation exercise modality on the muscle function of the quadriceps femoris and knee functional abilities in patients who underwent ACL reconstruction. We hypothesized that the rehabilitation exercise modality using NMES and BFR would enhance the quadriceps femoris and knee functional abilities in patients who underwent ACL reconstruction more than the general rehabilitation exercise modality.

## 2. Materials and Methods

### 2.1. Participants

**Figure 1:**
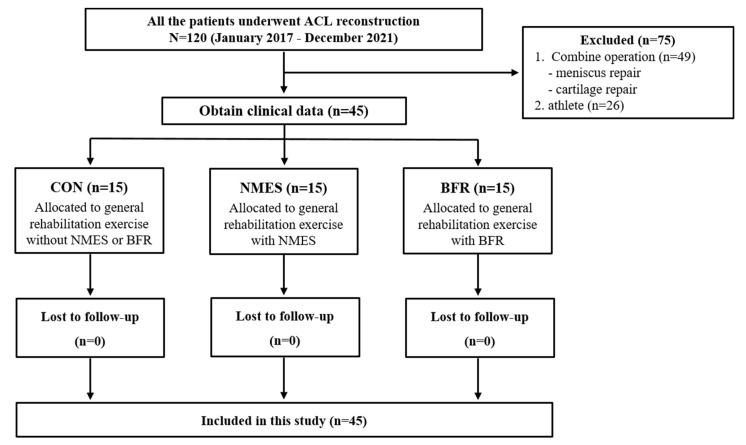
CONSORT flow chart. ACL, anterior cruciate ligament; CON, control; NMES, neuromuscular electrical stimulation group; BFR, blood flow restriction group.

Our study was conducted retrospectively using the test results of those who participated in the rehabilitation exercise process after ACL reconstruction from January 2017 to December 2021 by the same orthopedic knee surgeon at K hospital and M hospital. During the study period, one orthopedic knee specialist and a rehabilitation team moved to the hospital in July 2019 and used the same surgical method and rehabilitation exercise process to perform the same rehabilitation exercise. The present study included 45 Korean patients (age: 28.7 ± 8.2 years (34 males: 29.4 ± 8.5 years and 11 females: 26.8 ± 7.3 years)) who underwent cruciate ligament surgery using the same method via an orthopedic medical doctor. The CONSORT flow chart is illustrated in Figure 1. They were equally assigned to a control group (CON, n = 15) to undergo general rehabilitation exercise, an NMES group (n = 15), and a BFR group (n = 15) according to their age, sex, height, and weight. The CON consisted of subjects who underwent general rehabilitation from January to December 2017, and it was selected in consideration of age, gender, height, and weight. The NMES group consisted of subjects who applied the general rehabilitation and MNES from January 2018 to June 2019 at K Hospital. The BFR group consisted of subjects who applied the general rehabilitation and BFR from January 2020 to December 2021 at M Hospital.

Patients who underwent meniscus repair for a meniscus tear or cartilage repair for cartilage defects and elite athletes were excluded from the present study.

All participants received information about the purpose and process of the study, including possible side effects, and consent was obtained. Because all participants completed the study, all data were used in the analyses. As shown in Table 1, there were no significant differences in participants’ characteristics between the groups. All procedures were performed according to the ethical standards of the responsible committee on human experimentation and the Declaration of Helsinki. The study was approved by the Institutional Review Board of Konkuk University (7001355-202206-E-172) in Korea and was conducted according to the Declaration of Helsinki.

### 2.2. Study Design

**Figure 2:**
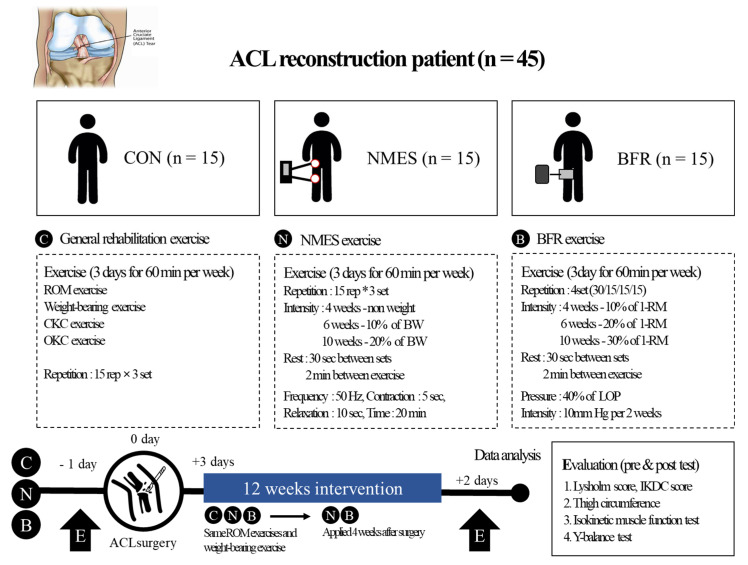
Study design. CON, control; NMES, neuromuscular electrical stimulation group; BFR, blood flow restriction group; CKC, closed kinetic chain; OKC, open kinetic chain; RM, repetition maximum; SBP, systolic blood pressure; IKDC, International Knee Documentation Committee; ROM, range of motion; ACL, anterior cruciate ligament.

The present study design is illustrated in Figure 2. All participants who underwent ACL reconstruction were divided into three groups: CON (n = 15, underwent general rehabilitation exercise), NMES (n = 15, underwent rehabilitation exercise with NMES), and BFR (n = 15, underwent rehabilitation exercise with BFR). All participants underwent a pre-test, a 12-week rehabilitation exercise program applied to each group, and a post-test.

In the present study, pre-tests were performed after being diagnosed with an ACL rupture by an orthopedic surgeon. The pre-test was completed one day before the ACL reconstruction, and the post-test began two days after the last rehabilitation exercise program. On the test day, all participants visited the Sports Medical Center at K and M hospitals and were tested after stabilization. All participants were measured for height and weight and evaluated on a subjective questionnaire (Lysolum score, IKDC subjective score). Then, to measure the circumference of the thigh, all participants were measured by relaxing in a supine position and measuring at the 5 cm and 15 cm points on the thigh. Then, muscle strength and endurance were measured to evaluate the isokinetic muscular function. For a dynamic evaluation of balance, the Y-balance test (YBT) was measured as an anterior reach, a posteromedial reach, and a posterolateral reach.

During the rehabilitation exercise session, a 12-week rehabilitation exercise program was conducted for all participants of each group three days after ACL surgery. The rehabilitation exercise program was performed three days per week for 12 weeks. The general rehabilitation exercise session consisted of exercises to enhance range of motion (ROM exercise), weight-bearing exercise, closed kinetic chain (CKC) exercise, and open kinetic chain (OKC) exercise. The ROM exercise was designed to promote the recovery of full ROM within six weeks. The ROM exercise was performed passively for knee flexion and then continued with active assistance. In addition, from week 4, an active motion was performed to achieve complete ROM. The ROM exercise increased the control of the pain and swelling gradually. Then, weight-bearing exercises were performed with the knees completely extended, such as weight shift exercises. From week 3, all patients were trained to walk normally as much as possible. We recommended that the patients wear braces until week 12. To ensure the complete extension of the knee, the angle of the brace was fixed at 0° until week 2; the patients were permitted to adjust the brace to their desired angle after week 2. The CKC exercises were initiated two to three weeks after surgery, such as wall squat, mini squat, half squat, and lunge, step-up exercise, and adding a gradual increase in weight from week 9. The exercise was performed in three sets of 15 repetitions. The OKC exercises for the quadriceps femoris were performed two to six weeks after surgery without weight bearing. From week 7, OKC exercise training with increased weight was performed at a limited angle of 90–45° flexion and was performed without angle restriction until three months after surgery. In addition, the OKC exercises for the hamstring muscle were initiated four weeks after surgery, such as prone active curl and standing active curl exercises, and a gradual increase in weight was added from week 9. The exercise was performed in three sets of 15 repetitions.

The NMES group and BFR group performed the same ROM exercises and weight-bearing exercises as the CON group. The NMES and BFR were applied four weeks after surgery. The NMES group performed the exercise by applying NMES when performing the same CKC exercise and OKC exercise as the CON group. The NMES group used the Kneehab device (Bio-Medical Research Ltd., Galway, Ireland) proposed by Feil et al. [28] with the general rehabilitation program. The Kneehab is an NMES device that wraps around the thigh and locates an array of four large electrodes over the quadriceps muscle. For the applied method, the frequency was 50 Hz, the contraction time was 5 s, the relaxation time was 10 s, and the duration time was 20 min.

The BFR group performed the exercise by applying BFR when performing the same CKC exercise and OKC exercise as the CON group. The BFR group used the Smart Cuffs device (Smart Tools Plus, OH, USA) with the general rehabilitation program. The BFR application method was the method proposed by Hughes et al. [29]. The intensity of exercise was started by setting a limit of 10–30% of the one-repetition maximum (1-RM) and was gradually increased. The BFR was achieved using hand-pumped blood pressure cuffs. The pressure was applied at 40% of the systolic blood pressure (SBP), and it was increased by 10 mmHg per two weeks.

Before and after the 12-week rehabilitation exercise session, each participant’s anthropometry (height and weight), Lysolum score, IKDC subjective score, thigh circumference (5 cm and 15 cm above the femur), isokinetic muscular function (strength and endurance), and balance (Y-balance anterior, posterior medial, and posterior lateral) were measured.

All testing procedures and rehabilitation exercise sessions were performed in the Sports Medicine Center at K and M hospitals.

### 2.3. Anthropometry (Height and Weight)

Height and weight were measured using a height and weight measuring instrument (BSM330, Inbody, Seoul, Korea). All participants wore lightweight clothing and were asked to remove all metal items from their bodies.

### 2.4. The Lysholm Score and the International Knee Documentation Committee (IKDC) Subjective Score

The Lysholm score and the IKDC subjective score of all the participants were measured, which are both clinical evaluation items that show the patient’s subjective knee functional status.

The Lysholm score was designed to measure the symptoms and function of patients with ACL and meniscus injuries. It does measure functioning in daily activities slightly, but it does not measure the domain of functioning in sports and recreational activities. This questionnaire consists of eight items with a total score of 100 points, with a higher score indicating fewer symptoms and higher levels of functioning.

The IKDC score was designed to evaluate patients with ligament and meniscus injuries as well as knee disorders such as patellofemoral pain or symptoms and determine their function in daily life and sports activities. This questionnaire consists of seven knee symptom items, two sports activity items, and two function items, with higher scores indicating fewer symptoms and higher levels of functioning.

### 2.5. Thigh Circumference

The thigh circumference was measured using a tape measure (Balzer 80206F, Hoechstmass, Sulzbach, Germany). To measure the circumference of the thigh, all participants were asked to lie down with their legs shoulder-width apart. In a state in which the force was removed, 5 cm and 15 cm were marked from the patella to the femur, and the circumference of the area was evaluated with a tape measure.

### 2.6. Isokinetic Muscle Function

The isokinetic muscle function (e.g., strength and endurance) was measured using an isokinetic dynamometer (Biodex system IV, Biodex medical, NY, USA) in the quadriceps femoris and hamstrings. The peak torque was measured four times at an angular velocity of 60°/s to evaluate the muscle strength, and the total work was measured 10 times at an angular velocity of 180°/s to evaluate muscle endurance.

### 2.7. Balance

The YBT for measuring dynamic balance ability was measured using the Functional Movement System’s YBT Kit (Functional Movement Systems, Inc., Chatham, VA, USA). The YBT was measured in each of the following directions: anterior, posteromedial, and posterolateral. This test was performed thrice in each direction while the person was standing barefoot. The anterior reach, posteromedial reach, and posterolateral reach were all measured, and the best value was recorded.

### 2.8. Statistical Analysis

All statistical analyses were conducted using IBM SPSS Statistics for Windows, version 25 (IBM Corp., Armonk, NY, USA). The data are presented as a mean ± standard deviation. The normality of the distribution of all outcome variables was verified using the Shapiro–Wilk test. A two-way analysis (time × group) of variance with repeated measures of the “time” factor was used to analyze the effects of rehabilitation exercise programs on each dependent variable. Partial eta-squared (η^2^) values were calculated as measures of the effect size. If a significant interaction or main effect within time or between groups was found, the paired *t*-test or independent *t*-test was used. A priori power analysis was performed with G-power for the isokinetic muscular function parameter (peak torque of the quadriceps femoris) based on previous research [14], indicating that a sample size of 12 participants per group would be required to provide 90% power at an α-level of 0.05. We anticipated a more than 10–20% dropout rate and aimed for a starting population of 15. The level of significance was set a priori at *p* < 0.05.

## 3. Results

### 3.1. The Lysholm Score and the IKDC Subjective Score

**Figure 3:**
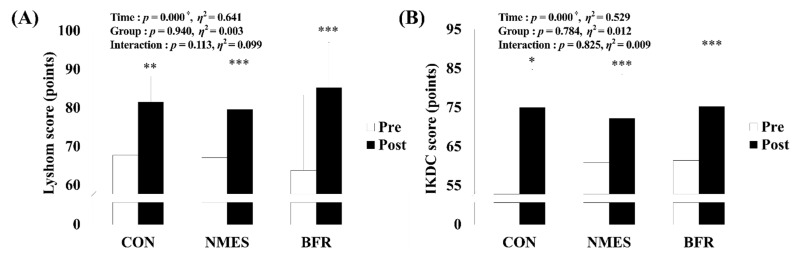
Changes in Lysholm score and IKDC subjective score via rehabilitation intervention in each group. (**A**) Change in Lysholm’s score. (**B**) Change in IKDC’s subjective score. CON, control group; NMES, neuromuscular electrical stimulation group; BFR, blood flow restriction group; IKDC, International Knee Documentation Committee. Significant interaction or main effect: † *p* < 0.05; Significant difference in each group via rehabilitation intervention: * *p* < 0.05, ** *p* < 0.01, *** *p* < 0.001.

As shown in Figure 3, a significant main effect within time was found for Lysholm score (*p* < 0.001, η^2^ = 0.641) and IKDC subjective score (*p* < 0.001, η^2^ = 0.529). Post hoc analysis found significant increases in Lysholm score (CON, *p* = 0.001; NMES, *p* < 0.001; BFR, *p* < 0.001) and IKDC subjective score (CON, *p* = 0.011; NMES, *p* < 0.001; BFR, *p* < 0.001) via intervention in all groups. However, there was no significant difference in Lysholm scores and the IKDC subjective scores between the three groups.

### 3.2. Thigh Circumference

**Figure 4:**
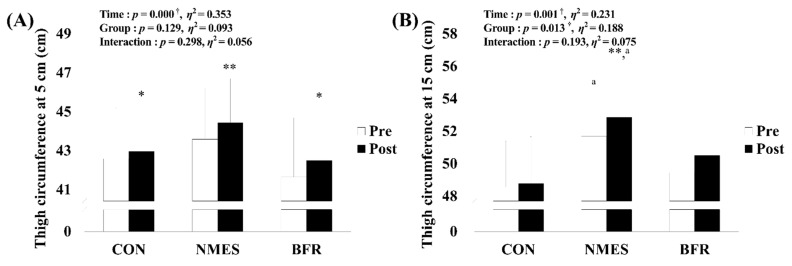
Changes in thigh circumference at 5 cm and 15 cm via rehabilitation interventions in each group. (**A**) Change in thigh circumference at 5 cm. (**B**) Change in thigh circumference at 15 cm. CON, control group; NMES, neuromuscular electrical stimulation group; BFR, blood flow restriction group. Significant interaction or main effect: † *p* < 0.05; Significant difference in each group via rehabilitation intervention: * *p* < 0.05, ** *p* < 0.01; Significant difference between groups each time: ^a^ *p* < 0.05.

As shown in Figure 4, a significant main effect within time for thigh circumference at 5 cm (*p* < 0.001, η^2^ = 0.353) and 15 cm (*p* = 0.001, η^2^ = 0.231) and a significant effect between groups for thigh circumference at 15 cm (*p* = 0.013, η^2^ = 0.188) was observed. Post hoc analysis found significant increases via intervention in all groups for the thigh circumference at 5 cm (CON, *p* = 0.021; NMES, *p* = 0.001; BFR, *p* = 0.028). However, the post hoc analysis found that NMES showed a significant increase in the thigh circumference at 15 cm via intervention (*p* = 0.007), and NMES showed a higher value in the thigh circumference at 15 cm before (*p* = 0.006) and after intervention (*p* = 0.002) than CON.

### 3.3. Isokinetic Muscle Function

**Figure 5:**
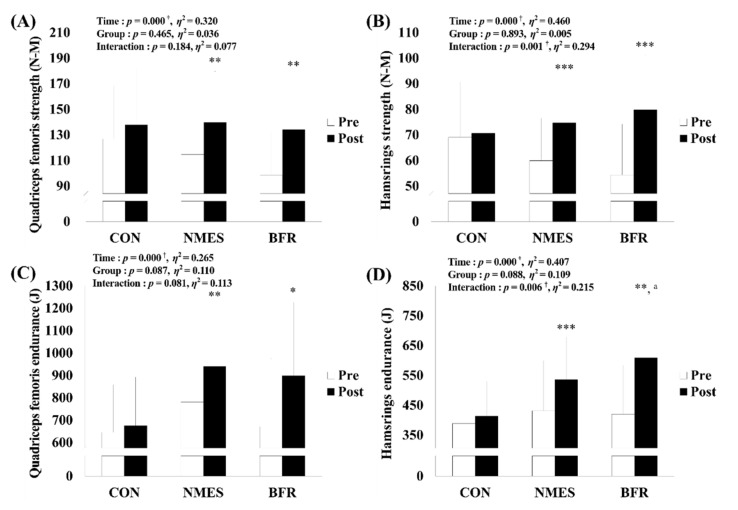
Changes in isokinetic muscle function via rehabilitation intervention in each group. (**A**) Change in quadriceps femoris strength. (**B**) Change in hamstring strength. (**C**) Change in quadriceps femoris endurance. (**D**) Change in quadriceps hamstring endurance. CON, control group; NMES, neuromuscular electrical stimulation group; BFR, blood flow restriction group, N-M; Newton–Meters, J; Joule. Significant interaction or main effect: † *p* < 0.05; Significant difference in each group via rehabilitation intervention: * *p* < 0.05, ** *p* < 0.01, *** *p* < 0.001; Significant difference between groups each time: ^a^ *p* < 0.05.

As shown in Figure 5, there was a significant interaction for hamstring strength (*p* = 0.001, η^2^ = 0.294) and hamstring endurance (*p* = 0.006, η^2^ = 0.215) as well as a significant main effect within time with respect to quadriceps femoris strength (*p* < 0.001, η^2^ = 0.320) and quadriceps femoris endurance (*p* < 0.001, η^2^ = 0.265). Post hoc analysis found significant increases via intervention in NMES and BFR in terms of the quadriceps femoris strength (NMES, *p* = 0.001; BFR, *p* = 0.001), hamstring strength (NMES, *p* < 0.001; BFR, *p* < 0.001), quadriceps femoris endurance (NMES, *p* = 0.001; BFR, *p* = 0.010), and hamstring endurance (NMES, *p* < 0.001; BFR, *p* = 0.001). In addition, the post hoc analysis found that BFR showed a higher value in hamstring endurance after intervention than CON (*p* = 0.003).

### 3.4. Balance

**Figure 6:**
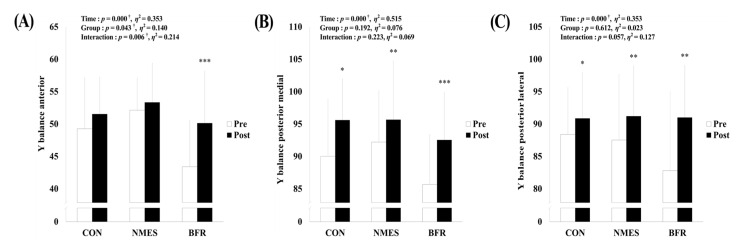
Changes in balance via rehabilitation intervention in each group. (**A**) Change in Y-balance anterior. (**B**) Change in Y-balance posterior medial. (**C**) Change in Y-balance posterior lateral. CON, control group; NMES, neuromuscular electrical stimulation group; BFR, blood flow restriction group. Significant interaction or main effect: † *p* < 0.05; Significant difference in each group via rehabilitation intervention: * *p* < 0.05, ** *p* < 0.01, *** *p* < 0.001.

As shown in Figure 6, there was a significant interaction for Y-balance anterior (*p* = 0.006, η^2^ = 0.214), and a significant main effect within time for Y-balance posterior medial (*p* < 0.001, η^2^ = 0.515) and Y-balance posterior lateral (*p* < 0.001, η^2^ = 0.353) was observed. Y-balance anterior significantly increased via intervention in BFR (*p* < 0.001), and Y-balance posterior medial (CON, *p* = 0.011; NMES, *p* = 0.003; BFR, *p* < 0.001) and Y-balance posterior lateral (CON, *p* = 0.010; NMES, *p* = 0.006; BFR, *p* = 0.008) significantly increased via intervention in all groups.

## 4. Discussion

In this study, the application of NMES and BFR after ACL reconstruction was shown to be effective for muscle function of the quadriceps femoris and knee functional abilities. In particular, it was effective in improving the muscle strength and muscular endurance of the quadriceps and biceps femoris, and YBT, a dynamic balance test, showed improved results. These results show that using NMES and BFR improves the muscle and knee function.

Among the results of this study, the Lysholm score and IKDC subjective score showed improvement after exercise compared to before surgery, and there was no difference between groups. A previous study that verified the effect of NMES after ACL reconstruction reported that there was no significant difference between groups in the IKDC subjective score, but it reported that the Lysholm score improved significantly compared to the CON group [28]. In addition, NMES used in rehabilitation exercise reported a moderate effect on self-reported patient outcomes at 12 weeks after surgery and is recommended to be included in rehabilitation after ACL reconstruction [30]. In addition, a previous study that verified the BFR effect reported an improvement in IKDC subjective score after eight weeks of BFR training as in this study [26] and reported that there was no difference between groups [31]. The subjective evaluation of knee function after ACL reconstruction is important, and the results of this study showed that the subjective evaluation of knee function after NMES and BFR was better than that before surgery.

In this study, as a result of comparing the thigh circumference before and 12 weeks after surgery, a significant change was found in the measurement results of the 5 cm thigh circumference of all groups. In the results of a previous study comparing three weeks of NMES training with a CON group of 19 healthy college students, when analyzing thigh circumferences after the exercise, a statistically significant increase was only observed in the left (non-dominant) limb [32]. After six weeks of BFR training, it was found that both the left and right circumferences of the healthy people were improved in the BFR group than in the CON group [33].

In another previous study, when healthy subjects were compared with the BFR group and the CON group after six weeks of BFR training, the thigh circumference improved by 3.5% in the BFR group, showing a statistically significant difference. BFR training is also helpful in the conservative treatment of diseases such as osteoarthritis, tendonitis, and muscle strains, and it is also a useful method for postoperative rehabilitation such as ACL reconstruction and arthroscopic surgery [34].

The results of our study showed significant improvements in quadriceps and hamstring strength and muscular endurance in the NMES and BFR groups.

As a result of a previous study of 12 weeks of rehabilitation exercise using NMES after ACL reconstruction, knee extensor muscle strength increased by 30.2% and 27.8%, respectively, at 90 and 180°/s at six months after surgery [28]. In another previous study, isokinetic torques of the knee extensor and flexor muscles were measured in a total of 49 subjects in the NMES group and the CON group, who were trained for six weeks by additionally applying NMES. Contrary to the results of our study, there was no significant difference, so it was reported that the application of NMES was not effective in improving muscle strength [35]. However, in several previous studies, NMES combined with rehabilitation exercise was seen to be more effective in improving quadriceps muscle strength than rehabilitation exercise alone. [30,36]. In addition, it was reported that NMES could be successful in improving quadriceps muscle strength when applied early in the postoperative rehabilitation period [37]. Therefore, the application of NMES is thought to help improve muscle strength.

Previous studies reported similar benefits of BFR training after ACL reconstruction, with declines in quadriceps muscle atrophy between days 3 and 14 postoperatively being less (11% vs. 22%) in patients who received BFR training during rest for 50 min per day for 10 days [38]. Ohta et al. [39] evaluated the changes in muscle strength of the quadriceps and hamstrings after applying BFR for 16 weeks after ACL reconstruction. The results were the same as in our study; the isokinetic strength of the extensors was greater in the BFR group compared to the CON group. The isokinetic strength in the knee flexors also showed better results in the BFR group than in the CON group.

As a result of the strength test in the previous study on the effect of BFR training in healthy subjects, there was a significant difference in extensor strength by 11% and muscular endurance by 15%. The flexor strength was improved by 11%, but there was no statistical difference between the two groups; meanwhile, muscular endurance was improved by 27%, and there was a significant difference. Such BFR training would greatly benefit patients with orthopedic diseases, as it provides the advantage of increased muscle strength without placing additional mechanical stress on inflamed or reconstructed tissues or joints [29,34].

The results of quadriceps muscle strength and muscular endurance improvement after BFR application were the same as the results of several previous studies [40,41], but there were previous studies that reported the same results of improved hamstring strength and muscular endurance [42], and there were studies that reported different results [40]. Among them, the BFR group showed improvement in extensor strength and endurance in the muscle strength test results after three weeks of training for patients with ACL reconstruction, but there was no difference in flexor strength and endurance [40]. This reported different results from our study. A previous study reported that hamstring muscles had many oxidative type I fibers, so BFR training would have been effective in improving flexor muscle function [43].

BFR training has also been shown to improve muscular endurance by improving muscle microvascular function and oxygenation [44,45]. BFR training can improve skeletal muscle hypertrophy and strength to a similar extent as heavy load resistance training, with a greater reduction in knee joint pain and effusion, leading to greater overall improvements in physical function. Therefore, BFR training may be more appropriate in the progressive limb loading phase of rehabilitation following surgery in patients who underwent ACL reconstruction [26]. Ultimately, the application of BFR can be presented as an effective method to strengthen the knee extensor and flexor muscles.

In the results of this study, the anterior reach of the YBT was significantly increased in the BFR group. YBT correlated with knee extensor and flexor peak torque in patients who underwent ACL reconstruction [46,47]. In addition, several previous studies have reported that the anterior reach of YBT was reduced after the general rehabilitation process in the ACL reconstruction group [48,49]. Therefore, an improvement of anterior reach after BFR application is an important result. This is because the results prove the effectiveness of BFR in patients with ACL reconstruction.

To prevent quadriceps muscle atrophy, which occurs frequently after ACL reconstruction, early strength increase can help to avoid quadriceps inhibition and atrophy and provide the most effective rehabilitation process for rehabilitation targets and return to sports [19]. Therefore, Park et al. [50] reported that aerobic exercise and strength training are important to maintain optimal physical strength due to problems such as a busy schedule or poor health. To enable continuous exercise for health management, it is essential to apply various new exercise methods such as NMES or BFR.

## 5. Limitations

The present study has the following limitations. First, this was a retrospective study. Therefore, there may be selection bias because the time of introduction of the equipment was different and the rehabilitation process was carried out in two hospitals. Second, the activity level before surgery was not considered. The results of the evaluations may have varied depending on the physical ability of the patients as well as their sex. Third, the three-month follow-up period was relatively short.

## 6. Conclusions

The present study confirmed that rehabilitation exercise programs using the NMES and the BFR are more effective rehabilitation methods for enhancing muscle function and balance ability in ACL reconstruction patients compared with general rehabilitation exercise. In the future, we need to conduct a study to confirm the mechanisms and effects of rehabilitation exercise programs using the NMES and the BFR on muscle hypertrophy and ligament strengthening in patients who underwent ACL reconstruction.

## Figures and Tables

**Table 1:** Participants’ characteristics.

Variable	CON (n = 15)	NMES (n = 15)	BFR (n = 15)	*p*-Value
Sex (male/female)	11/4	12/3	11/4	-
Age (years)	27.53 ± 8.43	29.13 ± 9.07	29.60 ± 7.60	0.780
Height (cm)	170.41 ± 76.83	173.47 ± 6.50	170.96 ± 7.63	0.495
Weight (kg)	76.83 ± 17.14	74.51 ± 11.72	70.79 ± 10.95	0.476
ACL leg (right/left)	6/9	8/7	4/11	-

Values are expressed as the mean ± standard deviation. CON, control group; NMES, neuromuscular electrical stimulation group; BFR, blood flow restriction group; ACL, anterior cruciate ligament.

## Data Availability

The data presented in this study are available on request from the corresponding author.
